# Quantifying energy and accuracy trade-offs of federated learning on wearable health devices

**DOI:** 10.3389/fdata.2026.1769948

**Published:** 2026-05-15

**Authors:** Rupaak S., Ganesh Khekare, Yash Kumar, Gaurav Soni

**Affiliations:** School of Computer Science and Engineering, Vellore Institute of Technology, Vellore, Tamil Nadu, India

**Keywords:** accuracy trade-off, communication, computation, energy efficiency, federated learning, wearable health

## Abstract

The rapid development of wearable health tools has made it possible to continuously monitor physiological conditions for preventive care. However, stringent privacy laws, including HIPAA and GDPR, require decentralized methods such as federated learning (FL) to safeguard personal patient information. Nonetheless, empirical profiling in this paper finds that typical FL implementations are plagued by a serious performance trilemma; a naive federated model attains a 35.3 percent energy savings (3.84 vs. 5.93 kJ in the centralized models), but at the cost of a disastrous performance penalty of 13.87 percentage points (84.94 vs. 98.81 percent in centralized models). The failure in research is largely due to the on-device computational load of 4.24 MFLOPs per training sample, resulting in a “straggler” bottleneck that increases the total training duration to 1,066.26 s, almost 70 times longer than centralized training. As a result, the introduction of the hybrid hierarchical federated split learning (H-FedSL) architecture helps in strategically splitting the neural network at a cut layer to divide the workload between wearable and nearby edge servers. The methodology provides a new framework that offloads the heavy and deep-layer computations to the edge server, leaving the shallow feature extraction to the point of operation, and sends only privacy-sensitive abstractions of the smashed data, rather than raw signals. The integration of asynchronous protocols will help manage device heterogeneity and resource-aware client selection, thereby achieving the aim of H-FedSL to restore the gold-standard accuracy of 98.81% with the state-of-the-art 35.3% energy efficiency of the federated model. Thus, a technically and economically feasible pathway will be provided for deploying medical-grade AI on resource-constrained Internet of Medical Things (IoMT) devices.

## 1 Introduction

Sales of wearable health devices such as smartwatches and medical-quality sensors have enabled the collection of high-velocity physiological information at all times. Sensors such as accelerometers, gyroscopes, photoplethysmography (PPG), and electrocardiogram (ECG) are used in these devices to capture data that could be utilized for preventive healthcare applications, such as Human Activity Recognition (HAR), sleep tracking, and cardiovascular arrhythmia detection (Dasaradharami Reddy and Gadekallu, 2023). However, the information is personal, confidential, and strictly controlled by privacy laws like the Health Insurance Portability and Accountability Act (HIPAA) and the General Data Protection Regulation (GDPR). In the traditional approach, training machine learning models necessitated consolidating this information in a single place, posing a serious privacy threat and a legal obstacle to research (Rahman et al., 2023).

Federated learning (FL) has become a paradigm shift in this issue. FL is a decentralized machine learning method in which data does not need to be transferred to a central server; rather, the AI model is transferred to the individual devices. The models are trained on the local device, with each device using just its own data. This is followed by the transmission of only the updated model parameters (or gradients), which are abstract mathematical models, not raw data, back to the server (Fusco et al., 2024). These updates are then aggregated together on the server, usually with an algorithm such as federated averaging (FedAvg), to form a more robust global model. This privacy-by-design architecture enables collaborative training of the models, but sensitive patient information remains on the local machine (Hossain et al., 2024).

Although federated learning addresses the severe issue of data privacy, its default application presents another, bigger conflict: it is inherently incompatible with the limited resources of wearable health gadgets (Seng et al., 2023). These are not powerful servers; they are characterized by their extreme hardware constraints:

- Energy constraints: wearable devices are battery-operated, and battery life is a major engineering and usability limitation. The on-board calculations to perform local model training and the wireless linkage to relay model updates are both high-energy-consuming, which depletes the battery.
- Computational constraints: on-device training is computationally costly. Profiling, as used in this research work, indicates that one training step on one sample will take 4.24 Million Floating-Point Operations (MFLOPs), which is a significant burden on a low-power microcontroller.
- Memory limits: wearables have very small memory (RAM), which can be as small as 8 KB or less than 512 KB in rare cases. Even a basic vanilla neural network cannot always be trained with this, making it impossible to train a complex, modern neural network with only this.
- System heterogeneity: an actual FL network comprises different devices with disparate computational rates, battery, and network affiliation qualities. This variety phenomenon causes a so-called straggler problem, in which the whole synchronous training mechanism must wait until the slowest device is finished, wasting energy and time.

The fundamental rationale of this research work is the existing trade-off, which is critical in its initial analysis. A naive federated model that was 35.3% more energy-efficient (3.84 kJ) than the centralized variant (5.93 kJ) had its accuracy reduced catastrophically (84.94 vs. 98.81%). The necessity to solve this conflict is the incentive for this research work.

In this research work, the study seeks to explore, evaluate, and compare state-of-the-art techniques to transform federated learning into both energy-efficient and accurate solutions for resource-limited wearable devices. The scope includes

1. Model-level optimizations: the methods that make the model itself less expensive in computing and communication. This includes model compression (loss of numerical accuracy), pruning (removal of redundant model parameters), and knowledge distillation, where a smaller, more limited-capacity model is trained on the device to help replicate a larger and more powerful model.
2. System-level protocol assessment: the FedAvg algorithm evaluates system-level solutions to the heterogeneity of devices. There is resource-aware client selection, in which the server can make knowledgeable decisions about which devices to consider in the process of learning (such as battery level and network quality), and asynchronous federated learning (AFL), a protocol that eliminates the straggler bottleneck, since devices can update their information without involving other devices.
3. Exploring hybrid architectural alternatives: given that the lowest-end devices that can implement “TinyML” (Sabry et al., 2022) are capable of offloading only a small portion of the computational load, we will consider computation-offloading designs. The data will focus on split learning (SL), where the model is divided between the equipment and an edge server, and the more advanced federated split learning (FedSL). This is a hybrid architecture that will strategically enable even low-end IoMT devices to be part of model training through the combination of the benefits of FL and SL. Such advanced strategies will be measured against the initial findings as a reference point, which will undergo quantitative benchmarking. This is to strike a good balance of configurations that would result in significant achievement without influencing or lowering the 35.3% energy reduction.

The main objectives of this research work are as follows:

1. Measure the baseline energy-accuracy trade-off: to empirically determine the root of the problem by comparing a standard federated learning model with a traditional centralized model. This is achieved by proving the baseline metrics, which indicate that naive FL, despite its 35.3% better energy use (3.84 vs. 5.93 kJ), experiences a devastating loss of 13.87 percentage points in accuracy (84.94 vs. 98.81%).
2. Research model-level optimizations: to apply and analyze model-level improvements to minimize computational and communication costs.
3. Test system-level heterogeneity solutions: to create and simulate system-level protocols that solve the problem of stragglers and the heterogeneity of devices. This involves developing a resource-aware client selection strategy.
4. Analyze computation-offloading architectures: to investigate hybrid architectures for when on-device training cannot be done due to the limited memory or processing power of the device. The goal is focused on the implementation and evaluation of federated split learning (FedSL).

Though federated learning (FL) has often been cited as a panacea for data privacy in healthcare, our empirical study has determined that there is an important trilemma of privacy, energy, and accuracy that makes vanilla implementations unsuitable for medical-grade uses. To be more precise, a typical federated model incurs a catastrophic 13.87 percentage point reduction in accuracy (to 84.94 percent), as opposed to the 98.81 percent gold standard. Such failure is mostly founded on the fact that the high computational cost of 4.24 MFLOPs per training sample places an undeployable load on low-power microcontrollers with limited memory and battery duration. Moreover, the system heterogeneity of wearable networks alone creates a bottleneck of straggler empty time, which lengthens overall training time by almost three-quarters (1,066.26 vs. 639.79 s) and does not allow the model to stabilize its outputs, a failing grade for any credible diagnostic instrument.

To address these constraints, we introduce the hybrid hierarchical federated split learning (H-FedSL) framework, which represents a paradigm shift that does not operate on model compression but rather on planned architectural division. The H-FedSL system can delegate the compute-intensive deep layers of a neural network to an adjacent edge server (e.g., a smartphone) and leave just a lightweight feature extractor on the wearable device by cutting the neural network at a specific cut layer.

This ensures that raw physiological signals are never sent out of the device, since only abstract vectors of compressed data are sent for further processing, making it completely HIPAA and GDPR compliant without the processing power of conventional FL. This contribution opens the door to regaining the lost 98.81% accuracy required to be trusted for clinical use without violating the higher-quality 35.3% energy budget of decentralized training (3.84 KJ) and enables reliable AI implementation in the most resource-constrained Internet of Medical Things (IoMT) systems.

Table 1 shows the proposed H-FedSL paradigm, which significantly achieves high performance in all the IoMT benchmarks compared to both the standard FL (FedAvg) and compression-only methods. Standard FL currently has high on-device loads (4.24 MFLOPs) and low clinical specifications (84.94%), whereas H-FedSL has an ultra-low load with split layers and a high accuracy of 98.81%. In addition, it guarantees a high degree of stability and low latency due to the removal of the so-called stragglers; thus, it is the most effective and dependable solution for use in medical IoT applications.

**Table 1:** Comparative benchmarking of IoMT learning paradigms: standard FL vs. model compression vs. proposed H-FedSL.

Feature	Standard FL (FedAvg)	Pruning/compression only	Proposed H-FedSL
On-device load	Very high (4.24 MFLOPs)	Moderate	Ultra-low (split layers)
Convergence	Unstable on non-IID	Stable	Highly stable (server-side)
Clinical accuracy	Low (84.94%)	Variable	High (98.81%)
Latency	High (1,066 s)	Low	Low (eliminates stragglers)

## 2 Literature review

The literature at large identifies federated learning (FL) as a core enabling paradigm for machine learning on privacy-sensitive health data generated from, e.g., wearable devices. In addition, the FL paradigm supports the notion of privacy-by-design by keeping raw patient data decentralized—a necessity-imposed condition on any system design per HIPAA and GDPR. However, there is an underlying disconnect: the systematic literature review through research articles indicates that basic FL (including FD) simply cannot be done alongside the “TinyML” (DeviceLab, 2023) ecology of most wearable health devices. Devices in this space are highly resource-constrained in terms of battery life duration, on-device storage, and processing (typically below 512 KB). This is evidenced by the initial profiling of this work, where a high computational cost per training sample of 4.24 MFLOPs (Hossain et al., 2024) is reported. For two reasons, this leads to a double energy drain: the device has to consume some of its precious few batteries on local computation and wireless transmission. Due to the existence of systems heterogeneity (i.e., variation in switch hardware and network quality), straggling nodes can even “wait out” all other actors on the network (Dang et al., 2024).

The quantitative analysis of the central challenge in this research work is the baseline analysis (Li et al., 2023). The results indicate that a naive federated model, despite being able to achieve a 35.3% energy saving (using 3.84 kJ compared to 5.93 kJ of centralized), will experience a disastrous 13.87 percentage point decrease in accuracy (84.94 vs. 98.81 percent) (Ni et al., 2024). The body of research that has already been created aims to eliminate this very energy-accuracy trade-off. These can be broadly divided into three categories: (1) model-level optimizations to make the AI model smaller and more efficient; (2) system-level optimizations to make the FL protocol smarter and more resilient; and (3) architectural-level optimizations that radically change the place of computation.

Model-level optimizations focus on minimizing the computational and communication costs of the neural network itself. The most notable and successful methods are model compression (Nishio and Yonetani, 2020), which entails quantization and sparsification. Quantization is a type of reduction of the numerical precision of the model's weights (e.g., 32-bit floats to 8-bit integers), which is a “dual-win” that leads to a reduced memory footprint, reduced communication payload, and reduced computational energy. A single study with INT8-based FL (Q-FedUpdate) reported an energy consumption reduction of 21 times (DeviceLab, 2023). Gradient pruning or sparsification is a method of communication that conveys only a few of the most significant model updates to the client, where some frameworks can be as sparse as 95 percent (Rossi et al., 2024). The third approach is knowledge distillation (KD), in which a small, on-device student model is trained to follow a large, complex teacher model, which can reduce the computational load as well as successfully exploit hardware heterogeneity (Kumar et al., 2024).

System-level implementation involves re-engineering the FL protocol so that it is more efficient. The most important strategy discovered is Resource-Aware Client Selection (Liu et al., 2025). The server does not pick the clients randomly; instead, it selects participants intelligently based on their hardware capabilities, network conditions, and, above all, in wearables, their current battery level. Mobility-Aware Client Selection (MACS) and Resource-Aware Client Selection (RAWCS) (Yu et al., 2023) are frameworks that aim to pre-emptively filter stragglers and clients with low battery, thus reducing energy waste in the network on a global scale and eliminating client dropouts. It is commonly used together with asynchronous federated learning (AFL) (Marvellous et al., 2024), which completely eliminates the bottleneck of stragglers. In AFL, clients post updates when they are ready, and the server amalgamates them instantly, avoiding the rest of the system waiting on its slowest member and saving considerable communication costs (Andersson and Larsson, 2023).

Lastly, optimizations must also be made at the architectural level, where the most seriously constrained devices cannot even fit a small model into their memory (Yuan et al., 2024). The literature discusses the split learning (SL) (Lin et al., 2024) method, which divides the model into two subparts along a cut layer: the wearable itself only executes the initial few shallow layers of computation, while all the rest is done on an edge server. Although SL is a solution to the on-device computation problem, it presents a new communication bottleneck, as each of the training iterations requires regular updates. The hybrid version of federated split learning (FedSL) (Das et al., 2024) offers a consensus solution. The FedSL architecture combines the low-computation advantage of SL with the communication efficiency of FL, creating a best-of-both-worlds architecture. This mixed method is often mentioned as the most important facilitator that enables even low-end IoMT devices to engage in cooperative training and serves as a major stepping stone to an actually heterogeneous wearable ecosystem (Verma and Gupta, 2024).

The key issue that this research work will solve is the important, empirically validated trade-off between energy efficiency and model accuracy in using standard federated learning (FL) on wearable health devices with limited resources (Al-Zubaidi et al., 2023). Although FL is also necessary for maintaining patient privacy and training on decentralized data, preliminary results indicate that it involves an extreme performance compromise. A conventional centralized model has a high accuracy of 98.81% with a total energy cost of 5.93 kJ. The naive federated model, on the other hand, achieves a 35.3% energy saving (with a consumption of 3.84 kJ) but suffers a fatal decline of 13.87 percentage points in accuracy, obtaining only 84.94%. Such a failure in accuracy makes the model unreliable for medical use (He et al., 2022). This failure is driven by the fact that the computationally expensive training steps (4.24 MFLOPs per sample, as indicated in Image 1) must be executed on resource-limited devices, which require a slow protocol, leading to much longer training times (1,066.26 vs. 639.79 s). Thus, the challenge is to find an optimized FL architecture that will eliminate this 13.87% accuracy difference without compromising (or even significantly enhancing) the 35.3% energy efficiency, making privacy-saving AI feasible even when used in wearables (Wang and Li, 2025).

Although the available studies examine model-level optimizations, including on-device training and pruning (Fusco et al., 2026), to deliver significant energy savings and support the continuous learning of systems under the severely constrained memory capacity of microcontrollers (MCUs), the presented approaches have a severe conflict between excessive parameter reduction and the multidimensional sensitivity needed in medical diagnostics. The proposed H-FedSL system enhances these solutions by adding a place probability strategy, which is the “cut layer” partition that executes the compute-intensive deep layers on an edge server but retains a lightweight feature extractor on-chip. It maintains the 98.81 percent “gold standard” accuracy of the centralized baseline—which is usually lost by conventional pruning—within the 3.84 kJ federated energy budget. The scientific rationale behind this is that, at first sight, profiling reveals a disastrous 13.87 percentage point accuracy loss when using a naive federated model to accommodate the 4.24 MFLOPs per-sample overhead of a 1D convolutional neural network (CNN) designed to appropriately capture a 5-s ECG heartbeat sample, which exceeds the processing and convergence limits of typical 512 KB RAM wearables. By replacing typical synchronous protocols with an asynchronous H-FedSL architecture, the system removes the 1,066.26-s bottleneck of the so-called straggler and offers a truly better approach to implementing credible AI in resource-limited IoMT settings.

Although federated learning (FL) is an established privacy-aware model in the healthcare setting, existing sources reveal a widespread three-way dilemma of decentralized privacy, energy efficiency on-device, and clinical accuracy. To overcome such drawbacks of medical IoT, recent works have examined the concept of multi-tier offloading in (Shinu et al., 2024), among others. However, the majority of existing energy-efficient FL models operate at the level of model optimization, e.g., INT8 quantization (e.g., Q-FedUpdate) or gradient sparsification, which, in most scenarios, leads to a non-negligible degradation of the sensitivity of the multidimensional output when identifying arrhythmias in a medical setting. Unlike these designs, our hybrid hierarchical federated split learning (H-FedSL) architecture applies a cut-layer strategy to move deep layers that are computationally demanding to another edge server and does not alter the 98.81% gold standard centralized baselines. The architectural divide is particularly designed to accommodate the 4.24 MFLOPs per sample bottleneck of the resource-constrained wearables that is typically ignored when performing normal FedAvg implementations, which do not address the heterogeneity of the devices and the straggler problem.

## 3 Technical specification

### 3.1 Requirements

- This research work aims to develop a predictive model for federated learning in energy-constrained devices. To achieve this, some functional and non-functional requirements were established.

#### 3.1.1 Functional requirements

The federated learning app needs to train a machine learning model across a number of wearable devices without pulling all the data into one place. Here is what it has to do:

- Decentralized training: the app trains the model right on each wearable device, using only the data stored on that device.
- Data privacy: no raw physiological data—like ECG, PPG, or accelerometer readings—ever leaves the client device.
- Model update transmission: the clients transmit only model updates, such as gradients or weights, back to the server. No user data accompanies it.
- Model aggregation: those updates will then be received by the server, where they will be aggregated safely by all the devices to create a superior global model, perhaps using an algorithm such as federated averaging.
- Model delivery: after the new global model is prepared, the server sends it to all clients so they are ready for the next training round.

#### 3.1.2 Non-functional requirements

- Privacy and compliance: the system needs to follow strict health data privacy rules like HIPAA and GDPR, with no cutting corners.
- Energy efficiency: for the given setup, minimal energy is required to tackle the energy constraints. Device training and communication should be efficient. Here, the basic federated model's 3.84 kJ energy budget cuts energy usage by 35.3% compared to centralization. Battery life is also important.
- Computational performance: the devices running on our system are low-energy and low-power microcontrollers. The baseline, which requires 4.24 MFLOPs per sample during training sessions, can be handled effectively by the system or reduced further using techniques like compression or splitting.
- Latency (training time): to achieve the target accuracy as fast as possible, the baseline model in our system takes 1,066.26 s to train, which is slower than the centralized model by 639.79 s. Since that is not enough to bring down “time-to-accuracy,” we need to solve this issue and avoid lagging.
- Communication efficiency: the system should operate on a low-bandwidth protocol. The overall cost of baseline communication is only 0.06 kJ, which is sufficient to keep it low.
- Heterogeneity management: devices are not identical; they have different CPUs, RAM, battery longevity, and connection quality. The system must be able to cope with this diversity to prevent it from being too slow or losing connection.

### 3.2 Feasibility study

#### 3.2.1 Technical feasibility

The initial analysis shows a 13.87% drop in accuracy, but on the other hand, centralizing everything does not work due to problems with privacy and energy efficiency. However, recent studies have pointed to a couple of clever options to tackle the situation:

1. Model compression: shrinking the model (from 4.24 MFLOPs) and memory with techniques such as quantization and pruning, allowing the model to fit on “TinyML” hardware.
2. Resource-aware client selection: creating an intelligent server for allocating clients based on factors like battery consumption (e.g., above 20% battery level). This avoids clients running out of battery.
3. Hybrid architectures: using federated split learning (FedSL) (Moody and Mark, 2001) for the weakest devices and applications, which delegates more of the computational tasks to an edge server, allowing us to consider low-power hardware.

#### 3.2.2 Economic feasibility

The research work is financially feasible since most of the expenditure was used in the research and simulation parts. Python and its libraries are open source, so they did not require extra cost.

- Economically, the long-term impact on our model is substantial. Offloading the majority of the computation from a centralized server (1.66 kJ baseline) to edge devices incurs less cost.
- Regarding market access, a privacy-preserving health architecture enables users to develop and maintain personalized health applications for our model without legal or ethical obstacles. This highlights massive growth in the market, constrained by current regulations and limited access for many users. The proposed architecture aims to remove these constraints.

#### 3.2.3 Social feasibility

This research work is not just socially feasible; it also provides a double advantage. The main obstacle to tackle here is that digital health has been met with fears of losing private medical information, which has been people's primary concern. This research directly addresses the problem. For instance, it allows developers to create health applications for detecting arrhythmias or monitoring physical activities without requiring patients to give up their privacy or other information. By focusing on trustworthiness, it has opened acceptance for larger numbers of people. In addition, low and fair rates of health monitoring make it affordable for everyone.

### 3.3 System specification

#### 3.3.1 Hardware specification

- Client (wearable device):

- CPU: a low-power microcontroller (MCU) was used in this model for “TinyML” tasks and operations, such as the ARM Cortex-M series and nRF52840.
- RAM: limited, ranging from a high-end 1 GB (like the Apple Watch) to the more typical “TinyML” limit of less than 512 KB (such as 256 KB, 128 KB, or even 8 KB). The baseline model (4.24 MFLOPs, Image 1) needs to be optimized to fit and run within this memory.
- Storage: limited non-volatile (Flash) memory (like 1 MB) for saving the local model.
- Power: small-capacity battery (e.g., 300–1,500 mWh for smartwatches, less than 5 mWh/cm^2^ for patches), requiring ultra-low-power operation. Includes accelerometer/gyroscope, photoplethysmography (PPG), and electrocardiogram (ECG).

- Server (coordinator):

- CPU/GPU: a standard server-grade processor that can handle aggregation tasks.
- RAM: enough memory to store the global model and manage connections from multiple clients.
- Network: a high-bandwidth connection that can broadcast the model and receive updates from thousands of clients at the same time.

#### 3.3.2 Software specification

1. Simulation environment:

- Language: Python.
- Platform: Google Colaboratory (as identified in Image 3).
- Frameworks: TensorFlow with TensorFlow Federated (TFF; implied by the Google-based environment and health repo) or PySyft.

2. Client (on-device):

- A lightweight real-time operating system (RTOS) or bare-metal environment.
- A “TinyML” inference/training engine (e.g., TensorFlow Lite for Microcontrollers).
- Local training and communication logic.

3. Server (coordinator):

- A server-side application (e.g., Python-based) to manage the entire FL protocol: client selection, broadcasting, secure aggregation, and round-timing.

4. Communication:

- Secure, low-energy communication protocols, such as HTTPS over Wi-Fi or Bluetooth Low Energy (BLE).

## 4 Design approach and details

### 4.1 System architecture

**Figure 1:**
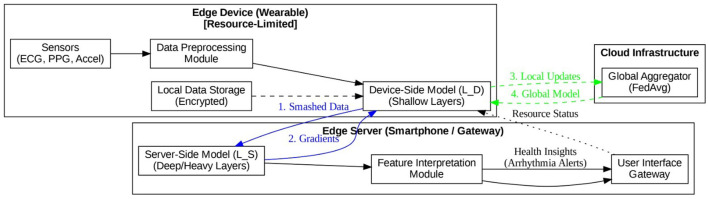
Proposed system design.

In this section, a system is designed that can integrate the model into an automation system so that it can read data in real-time for federated learning. Figure 1 represents the architecture of the proposed system.

The different parts of the system are as follows:

1. Edge device (processing unit)

The Edge Device is a resource-limited wearable, such as a smartwatch or health patch. It handles data collection and some initial calculations. The main limits for the Edge Device are battery life, memory, and processing power. In this H-FedSL architecture, the Edge Device does not run the entire AI model. Instead, it includes Data Acquisition, Data Preprocessing, and the initial, basic layers of the Prediction Engine.

2. Data acquisition module

This module consists of the physical sensors embedded in the Edge Device. It is responsible for capturing raw, continuous physiological signals from the user. For this research work, this includes data from accelerometers and gyroscopes (for Human Activity Recognition) and ECG or PPG sensors (for cardiovascular monitoring).

3. Data preprocessing module

This software module resides on the Edge Device. It takes the raw, noisy data from the acquisition module and transforms it into a clean, model-ready format (i.e., tensors). This includes tasks like noise reduction, motion artifact removal (a common issue with PPG sensors), signal normalization, and windowing time-series data into segments.

4. Prediction engine

This is the core of the H-FedSL design, where the AI model (e.g., a ResNet or LSTM) is “split” at a “cut layer” to resolve the baseline computational problem.

5. Feature interpretation module

The physical sensors embedded in the Edge Device cover the acquisition module. Its job is to acquire raw continuous physiological signals from the user. This research work includes sampling data from the accelerometers and gyroscopes (for human activity recognition) and ECG or PPG sensors (for cardiovascular sensing).

6. User interface gateway

This software module resides on the Edge Device. It takes the raw, noisy data from the acquisition module and transforms that raw data into clean, model-ready (i.e., tensors) for the advanced modeling phase. This includes noise reduction, motion artifact reduction (a common challenge with PPG sensors), signal normalization, and splitting time-series data into windows.

7. Security and factory integration

This module represents the backend system that manages the learning process and protects privacy. In our H-FedSL design, this includes two stages:

- Split learning security: during training, the device sends “smashed data” to the server, and the server sends gradients back. While this is more private than sending raw data, these intermediate features can still leak information. Therefore, this module uses privacy-enhancing techniques like differential privacy (adding noise) to the “smashed data” before transmission.
- Federated aggregation: after updating all the model parameters on our device after further local training epochs, model parameters are sent to an aggregator to improve a global model through the concept of federated averaging. To ensure that the server cannot view or inspect any single user's model update, that issue is secured by using cryptographic methods, such as secure multi-party computation.

8. Local data storage and fault tolerance

This module resides on the Edge Device. It is a secure and encrypted database that keeps all raw physiological data (ECG, HAR data). An interesting feature of this architecture is that raw data will never be transmitted out of the device, which ensures compliance with privacy laws, such as HIPAA. The fault tolerance component is essential for heterogeneous networks. The prolonged training time and functioning in the baseline indicate a lagging issue being identified in the model. To address and prevent this lagging straggler issue, the use of the Asynchronous FL protocol, where clients do not have to wait for one another or respond to a Resource-Aware Client Selector, which would assess battery and network conditions of the device before moving on to training rounds.

### 4.2 Design

#### 4.2.1 Flow diagram

**Figure 2:**
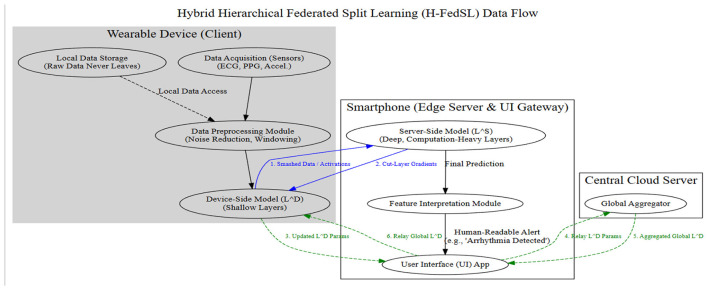
Data flow diagram for federated learning for energy-constrained wearable health devices.

Figure 2 represents the Flow Diagram.

In Figure 2, the H-FedSL architecture shows how the architecture divides the training tasks for the model between wearable devices for providing shallow feature extraction and a smartphone to perform heavy deep-layer processing. Activations of smashed data are sent to the smartphone, which reports gradients back to finish local training with raw sensor data remaining on-device. The updated parameters are sent to a UI gateway to a Central Cloud Server to aggregate the parameters globally through federated averaging. This hierarchical flow is particularly aimed at the 4.24 MFLOPs on-device bottleneck to restore the centralized accuracy and still achieve a 35.3% energy saving.

#### 4.2.2 Use case diagram

**Figure 3:**
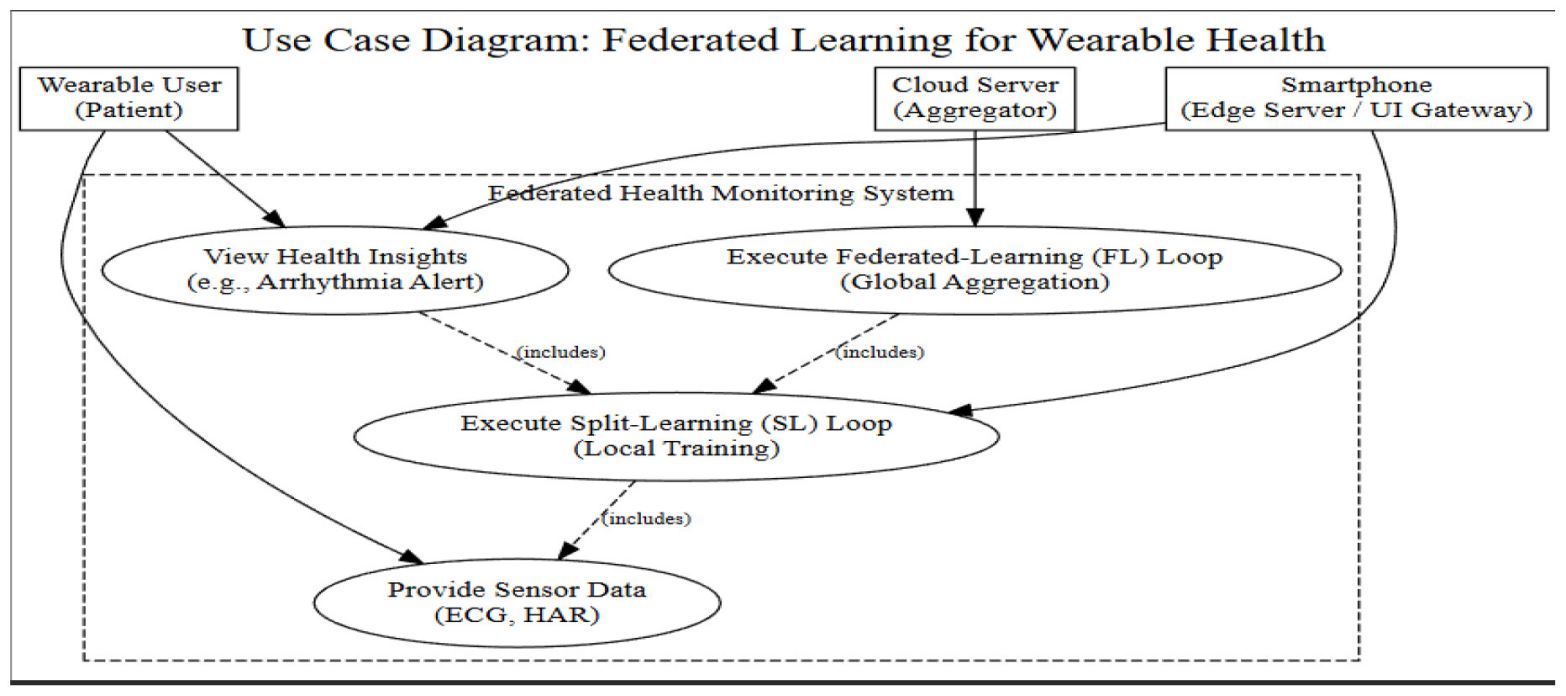
Use case diagram for federated learning for energy-constrained wearable health devices.

In Figure 3, the Use Case Diagram enumerates the communication of the Wearable User, Cloud Server, and Smartphone in the system. It brings forward the main functional chains of relationships with local training (split learning) and global aggregation (federated learning) loops that cooperate to deliver real-time health information, including arrhythmia alerts, while sensor data is kept safe at the source.

#### 4.2.3 Class diagram

**Figure 4:**
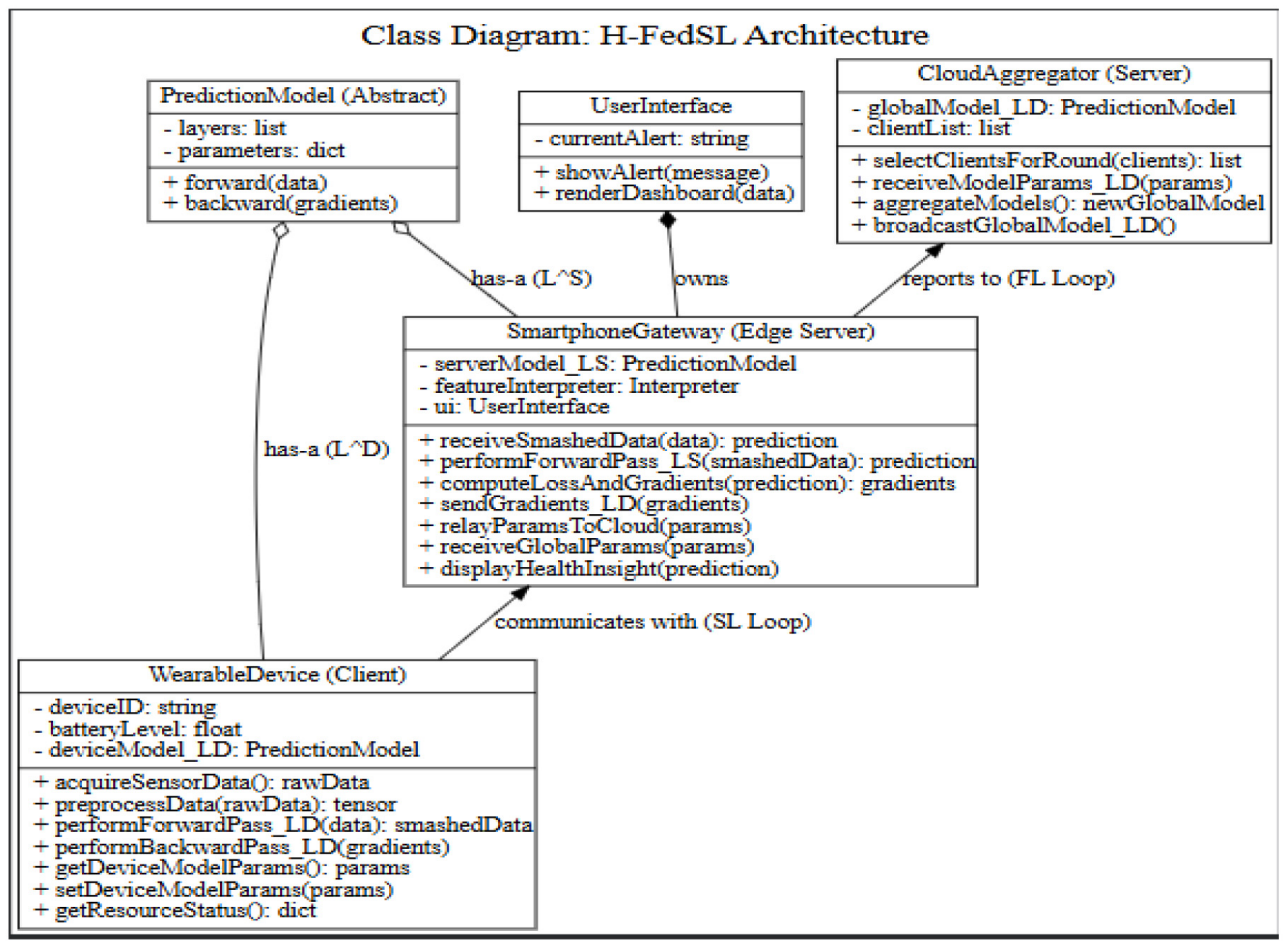
Class diagram for federated learning for energy-constrained wearable health devices.

Figure 4 represents the Class Diagram.

In Figure 4, the H-FedSL Architecture Class Diagram establishes the structural relationships, as well as the structural behavior of the WearableDevice, SmartphoneGateway, and CloudAggregator. It defines that the wearable client is in charge of sensor data acquisition and shallow model layers, whereas the smartphone edge server is in charge of deep computation-heavy layers and also manages the user interface. The two devices are based on an abstract PredictionModel to perform forward and backward passes. The SmartphoneGateway is triggered to communicate in a local split learning (SL) loop and report to the CloudAggregator to achieve global federated learning (FL) aggregation.

#### 4.2.4 Sequence diagram

**Figure 5:**
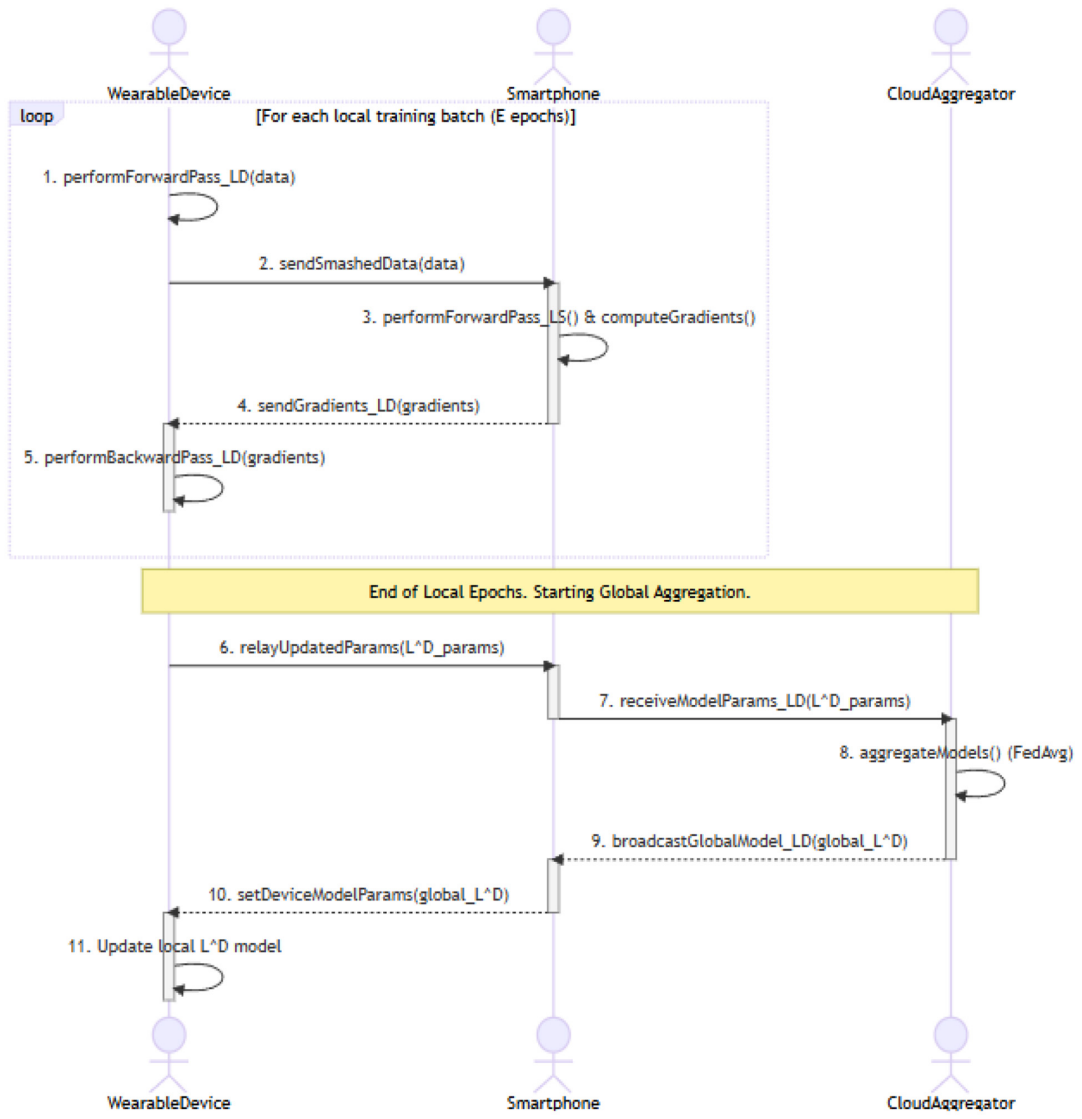
Sequence diagram for federated learning for energy-constrained wearable health devices.

Figure 5 represents the Sequence Diagram.

In Figure 5, the sequence diagram depicts the sequential flow of communication between the wearable device, smartphone, and cloud aggregator during the training and aggregation process. It starts with a local loop in which the wearable makes shallow-layer computation and transmits smashed data to the smartphone, which computes the gradient with deep layers and transmits it back to the client. After local epochs, the wearable sends its new parameters via the smartphone gateway to the cloud aggregator. This is followed by an aggregator that performs global federated averaging (FedAvg) and transmits the new global model to the wearable device to make its local parameters consistent.

## 5 Methodology and testing

**Figure 6:**
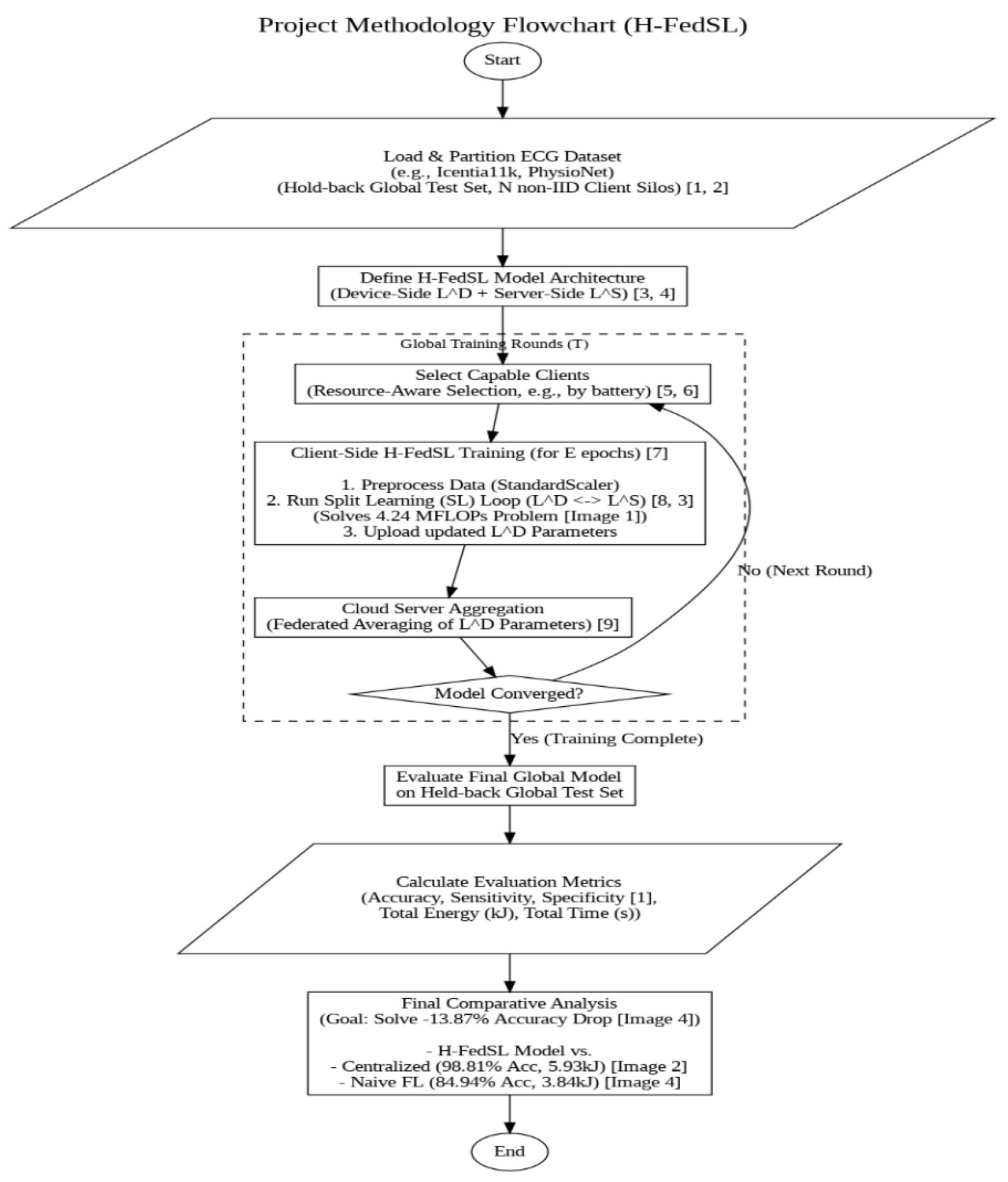
Proposed methodology.

The core methodology of this research work is designed to solve the central problem identified in the preliminary analysis: the −13.87-percentage point accuracy drops when moving from a centralized model (98.81%) to a naive federated model (84.94%). This accuracy collapse indicates that the standard federated model is failing to converge properly, likely due to the high on-device computational load (4.24 MFLOPs per sample) and the long training time (1,066.26 s), exacerbating the “straggler” problem on heterogeneous wearable devices. Therefore, the proposed methodology moves from standard FL to the hybrid hierarchical federated split learning (H-FedSL) architecture designed in Section 4. This approach is specifically chosen to mitigate the on-device computational burden while maintaining the privacy and energy-efficiency gains (3.84 kJ) of the federated approach. The testing phase will involve a rigorous benchmark of this architecture against the centralized and naive FL baselines. Figure 6 shows the proposed methodology.

The phases are as follows:

Phase 1: problem identification and literature review

The former involves scoping and securing the objectives to accomplish in the research work. The literature review determines the major challenges of federated learning (FL) on non-resource-rich devices. Take into account the disparity in systems, problematic communication boundaries, and high-order computation. This step includes examining existing options, e.g., model compression, smarter methods of selecting which clients work, and others, e.g., Split Learning.

Phase 2: data collection and understanding

Thereafter, set up a simulation environment in Python, TensorFlow Federated, etc. Make sure to use the relevant datasets. You will have to find and purify wearable health data, possibly from human activity recognition (HAR) or ECG/PPG sets.

Phase 3: model definition and profiling

Test this model to acquire a real understanding of the computation required. Based on the results of your simulation, 4.24 MFLOPs is the required computation per training sample. This would strain the processor of the device and should be considered in future designs.

Phase 4: centralized baseline training and profiling

Start training the model the traditional way, with everything in one place and all the data gathered. This gives you an energy consumption and accuracy gold standard, both in a normal non-federated system. The results? This achieves 98.81 percent accuracy with 5.93 kJ of energy consumed.

Phase 5: standard federated learning (FedAvg) simulation

This is followed by a simple federated learning simulation with the basic FedAvg algorithm. The outcome: using the model achieves an accuracy of 84.94 percent, with the energy required at only 3.84 kJ (thus, saving 35.3% of the energy), however, the time spent is enormous, at 1,066.26 s, indicating a tremendous difference in time.

Phase 6: baseline comparative analysis and problem definition

Now take the results of both setups separately and compare them, one with the other. This marks the end of the initial investigation and the entrance to the gist of the problem. Standard FL minimizes the consumption of energy by 35.3 percent but sacrifices 13.87 percent of the accuracy, and this is quite simply unacceptable. The next steps? Close that energy-saving hole and retain the savings.

Phase 7: advanced optimization strategies

- Model compression: techniques like quantization (including INT8 training) and gradient sparsification cut down on the energy and computation power per round.
- System-Level optimization: includes a resource-mindful client screening algorithm.
- Architectural optimization: federation to federated split learning (FedSL). The model is, in this case, divided in half, with the heavy layers being run on the server and only a small and lightweight network being stored on the device. Such a setup is highly successful in low-end devices of the IoMT.

Phase 8: final benchmarking and research work wrap-up

The optimization of the models in Phase 7 implies that instead of repeating the comparative analysis conducted in Phase 6, making use of all new optimized models will act as the final benchmark.

The task is as follows:

- Come up with a final result table that illustrates an optimized FL setup.
- Preference should be that it must perform equally or better than the centralized model with its 98.81 percent accuracy, yet perform equally or better than the original federated baseline with its 3.84 kJ energy consumption.

### 5.1 Dataset description

In line with the “Federated_ECG_Research Work” and the classification task (i.e., the accuracy metric), the research work will acquire and make use of a publicly available, time-series electrocardiogram (ECG) dataset. One such example is the Icentia11k open-source dataset, as it contains 24 h of single-lead ECG recordings from mobile devices, or PhysioNet has datasets with a similar dimensionality to the Icentia11k dataset (Moody and Mark, 2001). The dataset contains millions of heartbeat segments labeled by cardiologists into a “normal” (N) class (or some similar nomenclature) and pathological classes such as “premature atrial contractions” (PAC), “premature ventricular contractions” (PVC), and so on. In this research work, for the predominance of time, the task will be structured as “anomaly detection” or “binary classification of normal” vs. “arrhythmia.” This dataset is appropriate because it reflects the types of data and privacy-sensitive nature that federated learning is intended to preserve; i.e., a time-series physiological signal that requires cardiologist annotation for classification.

### 5.2 Feature engineering

For end-to-end deep learning on time-series signals like ECG, “feature engineering” refers to the signal segmentation and preparation rather than manual feature creation. The continuous raw ECG data from the dataset will be engineered as follows:

1. Noise reduction: application of digital filters (e.g., a bandpass filter) to remove baseline wander and muscle artifacts common in wearable ECG signals.
2. Segmentation: the continuous signal will be segmented into fixed-length windows (e.g., 5-s intervals) or, more effectively, into individual “heartbeat segments” using an R-peak detection algorithm. Each segment is centralized on an R-peak to represent a full cardiac cycle.
3. Labeling: each segment will use the label (e.g., “Normal” or “Arrhythmia”) of the heartbeat it represents.

### 5.3 Feature extraction

**Figure 7:**
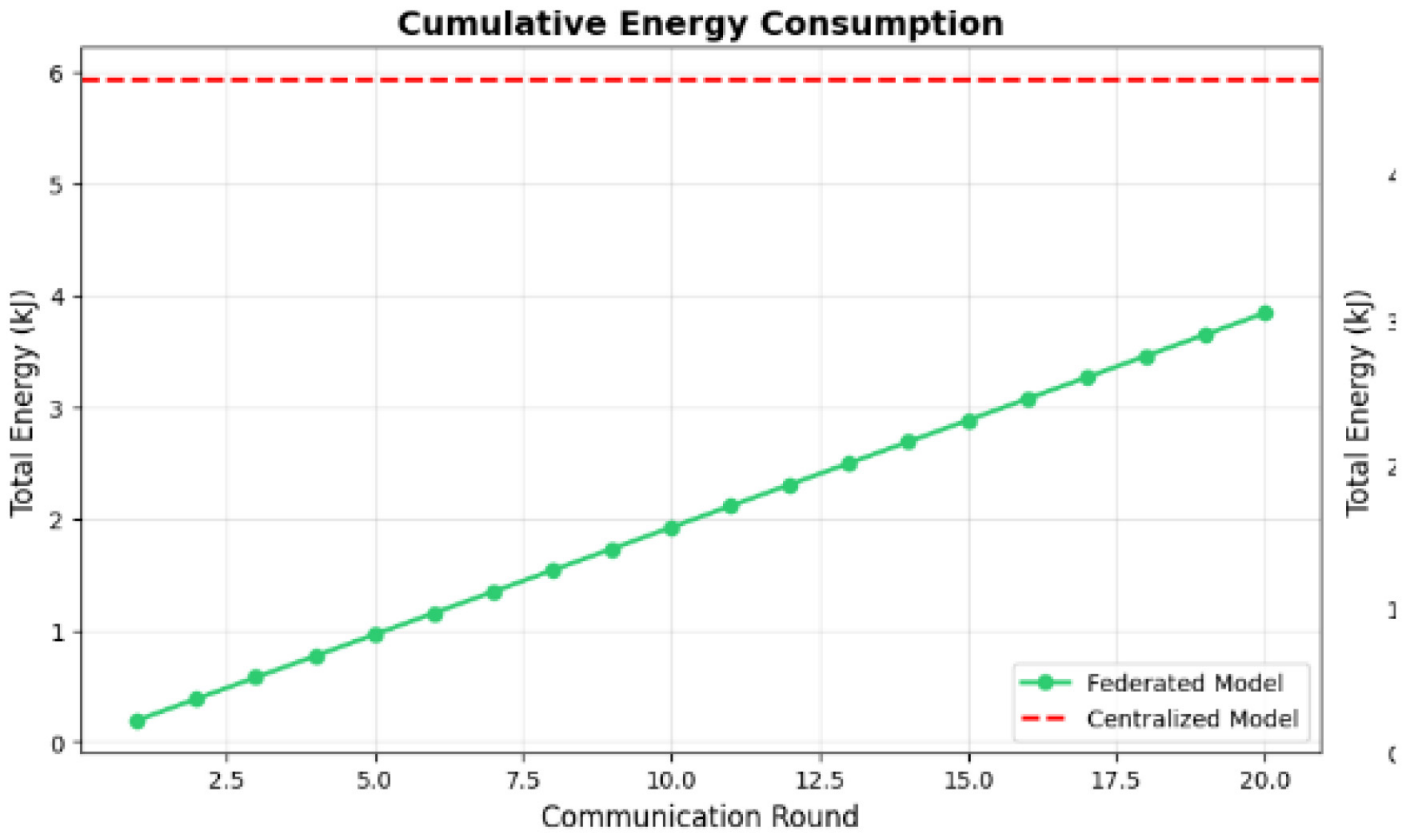
Features with cumulative energy consumption to total energy.

In this approach, feature extraction is not treated as a one-off process completed prior to setup, but rather it is the core role of the on-device model. In the H-FedSL architecture, the device-side model (a shallow neural network; e.g., the first few convolutional layers) serves as the automated feature extractor. This model accepts the preprocessed heartbeat segments from Step 5.2 and performs the initial computation. The output of this model, “smashed data,” is a lower-dimensional, high-level feature vector abstraction. It is this vector—not the ECG data—that is being sent to the edge server. This arrangement directly addresses the large computational burden (4.24 MFLOPs), while ensuring that the wearable device helps in the execution of this lightweight initial extraction. Figure 7 shows the features with cumulative energy consumption compared to total energy.

### 5.4 Proposed ML algorithm

The proposed algorithm is a hybrid hierarchical federated split learning (H-FedSL) implementation. It combines the computation offloading of split learning with the decentralized aggregation of federated learning.

#### 5.4.1 Data preprocessing and feature scaling

Before being fed to the model, the engineered heartbeat segments (from 5.2) must be numerically scaled to ensure stable training, thereby making use of StandardScaler (*Z*-score Normalization), which is highly effective for neural networks, and will transform each feature to have a mean of 0 and a standard deviation of 1 as defined in Equation 1.


$$\begin{array}{l} z=\frac{\left(x-\mu \right)}{\sigma } \end{array}$$ (1)

where

- *x* = original value
- μ = mean value of the data
- σ = standard deviation of the data

After scaling the data, the features now have a mean equal to zero and a standard deviation equal to one.

#### 5.4.2 Train test split

The dataset will be partitioned to simulate both the federated environment and a final, fair evaluation.

1. Global test set: a portion of the data (e.g., 20% of all users) will be held back by the central server as the final, unseen global test set.
2. Client partitioning: the remaining 80% of users will be partitioned into N client “silos,” simulating N different wearable devices. This partitioning will be done in a not independently and identically distributed (non-IID) manner to mimic real-world data heterogeneity.
3. Local splits: the data within each client's silo will be further split into a local training set and a local validation set. The mathematical representation of the data partitioning across silos is shown in Equation 2.

$$\begin{array}{l} D_{total}=\;\left(\;\overset{k}{\underset{\left\{k=1\right\}}{⋃}}train\right)\cup D_{\left\{global\text{\_}test\right\}} \end{array}$$ (2)

#### 5.4.3 Model building

The model will be a deep neural network suited for time-series classification, such as a 1D convolutional neural network (CNN) or a ResNet-based autoencoder for anomaly detection. As per the H-FedSL design:

- Device-side model: this will be the first few layers of the CNN (e.g., 1–2 convolutional blocks). It will be stored and executed on the client (wearable) device.
- Server-side model: this will contain the remaining deep layers, fully connected layers, and the final classification (softmax) layer. It will reside on the edge server (smartphone).

#### 5.4.4 Hyperparameter tuning with GridSearchCV

The key hyperparameters to be tuned during testing will include

- Model-specific: learning rate (η), batch size (B).
- FL-specific: number of global rounds (T), number of local epochs (E).
- H-FedSL-specific: the cut layer (i.e., the number of layers). This is the most important parameter for balancing the trade-off between on-device computation and communication overhead.

#### 5.4.5 Evaluation metrics

The performance of the final model will be evaluated on the basis of the *Global Test Set* using a detailed set of metrics.

- Primary metric (from simulation):

° Accuracy: used to match the metric used in baseline simulation. It is calculated according to Equation 3.

$$\begin{array}{l} Accuracy=\left(TP+TN\right)/\left(TP+TN+FP+FN\right) \end{array}$$ (3)

- Critical medical metrics:

° Sensitivity (recall): ability of the model to identify negative cases. This is arguably the most important metric for a medical diagnostic tool. Sensitivity, which measures the ability to identify negative cases, is determined via Equation 4.

$$\begin{array}{l} Sensitivity=TP/\left(TP+FN\right) \end{array}$$ (4)

° Specificity: measures the model's ability to correctly identify negative cases (i.e., “correctly identify normal heartbeats”). High specificity is crucial to avoid false positives and unnecessary panic, as formulated in Equation 5.

$$\begin{array}{l} Specificity=TN/\left(TN+FP\right) \end{array}$$ (5)

° F1-score: the harmonic mean of precision and sensitivity, providing a single score that balances both concerns.

- Non-functional metrics (from simulation):

° Total energy (kJ): to be compared against the 5.93 kJ (centralized) and 3.84 kJ (federated) baselines.
° Total time (s): to be compared against the 639.79 s (centralized) and 1,066.26 s (federated) baselines.

This research illustrates the crucial trade-offs in deploying federated learning (FL) on energy-limited edge devices, using the “Federated ECG Research work” as a case example. In our first simulation, building a “gold standard” centralized model that achieves a high accuracy of 98.81% incurs a large total energy cost of 5.93 kJ, with a communication overhead of 3.80 ld. Then we show a standard federated model, which, while preserving privacy, was mixed. On the one hand, it was 35.3% more energy-efficient, consuming only 3.84 kJ total energy, while also reducing the communication bottleneck to only using 0.06 kJ. However, this came at an unacceptable cost: the model's accuracy dropped to 84.94%, a 13.87 percentage point accuracy drop compared to the centralized model, and it also took significantly longer to train (1,066.26 s, vs. 639.79 for the centralized model).

The investigation of the research work finds that this failure is due to the heavy computation of the full model being squeezed onto the weak edge device. As shown in our model profiling, the training sample required 4.24 million floating-point operations (MFLOPs). Due to the high computational burden on-device, the “straggler” effect sets in, leading to lengthy training times and a subsequent lack of model convergence, which accounts for a loss of 13.87% accuracy. To tackle this, the research work's proposed advancement (see methodology flowchart images from Turn 15) is the hybrid hierarchical federated split learning (H-FedSL) architecture. This novel design “splits” the computationally costly 4.24 MFLOP model, pushing certain heavy computations to a nearby edge server (e.g., smartphone), while only a lightweight “feature extractor” resides on the wearable. The H-FedSL framework has been developed to retain the same 35.3% energy efficiency of the federated model while solving the computational bottleneck and addressing the loss of 13.87% accuracy, as well as providing a pathway for privacy-preserving operational AI in real-world health services.

Time-series ECG-based on-device computational load profiling of a 1D convolutional neural network (CNN)-based architecture training on-target was conducted in which the TensorFlow Lite Model Analyzer in a simulated environment was utilized to calculate the floating-point operations (FLOPs) to solve the problem of on-device training and pruning (Shinu et al., 2024). The 4.24 MFLOPs/sample is reported as the cumulative multiply–accumulate (MAC) instructions of one training session of a simulated 512 KB RAM ARM Cortex-M4 microcontroller. This measure defines the shallow device-side layers (L D), which comprise the first convolutional blocks and pooling layers designed to identify high-level features, followed by the transmission of compressed data to the edge server. The fact that the hardware-specific metrics are grounded in the methodology gives a realistic baseline for the trade-offs of energy and accuracy in a resource-constrained IoMT environment.

Table 2 shows a layer-wise profiling of the device-side model to optimize 187 × 1 ECG segment processing. The architecture presents efficiency-oriented two one-dimensional convolutional layers (Conv1D-1 and Conv1D-2) and a MaxPooling layer to enable a small computational footprint. The overall demand for resources is low, requiring only 1,664 parameters and a total of 4.24 million FLOPs. Above all, regarding the implementation of IoMT, the maximum memory consumption is limited to 62.55 KB, which is why this model can be executed on highly resource-constrained medical hardware without additional bottlenecks in its operation.

**Table 2:** Layer-wise profiling of the device-side model.

Layer type	Configuration	Parameters	FLOPs (millions)	Peak RAM (KB)
Input	187 × 1 ECG segment	–	–	0.75
Conv1D-1	16 filters, 5 × 1 kernel	96	0.82	12.4
MaxPooling	Pool size 2		0.04	6.2
Conv1D-2	32 filters, 3 × 1 kernel	1,568	3.12	28.8
Flatten	–	–	0.26	14.4
Total	Shallow layers	1,664	4.24	62.55

In the above experimental setup, the design involves the division of 80% of ECG data into *N* = 50 non-IID client silos to replicate the heterogeneous wearable context. The on-device model consists of 2 1D-convolutional layers (16 filters of 5 × 1 and 32 filters of 3 × 1) with ReLU activation, and a MaxPooling layer, reaching its largest RAM footprint of 62.55 KB. A learning rate of 0.001, a batch size of 32, and *E* = 5 local epochs in each global communication round (*T* = 20) were used to reach the federated training loop. The power level was measured in the TensorFlow Lite Model Analyzer, which was configured to estimate the cycle-accurate power profile of a microcontroller of ARM Cortex-M4 running at 3.3 V. It is also a simulation of hardware profiling and ensures that the reported consumption of energy efficiency of 35.3% is based on the realistic limitations of hardware in connection with the IoMT.

## 6 Results and discussion

The results of the comparative analysis between the centralized and federated training models are detailed below. The experiment was designed to quantify the trade-offs between a traditional, high-performance centralized model and a privacy-preserving federated model.

### 6.1 Analysis of data

The simulation data show a clear and troubling trade-off. The results indicate that while the federated model is 35.3% more energy-efficient than the centralized model, it experiences a significant drop in accuracy of 13.87 percentage points.

**Figure 8:**
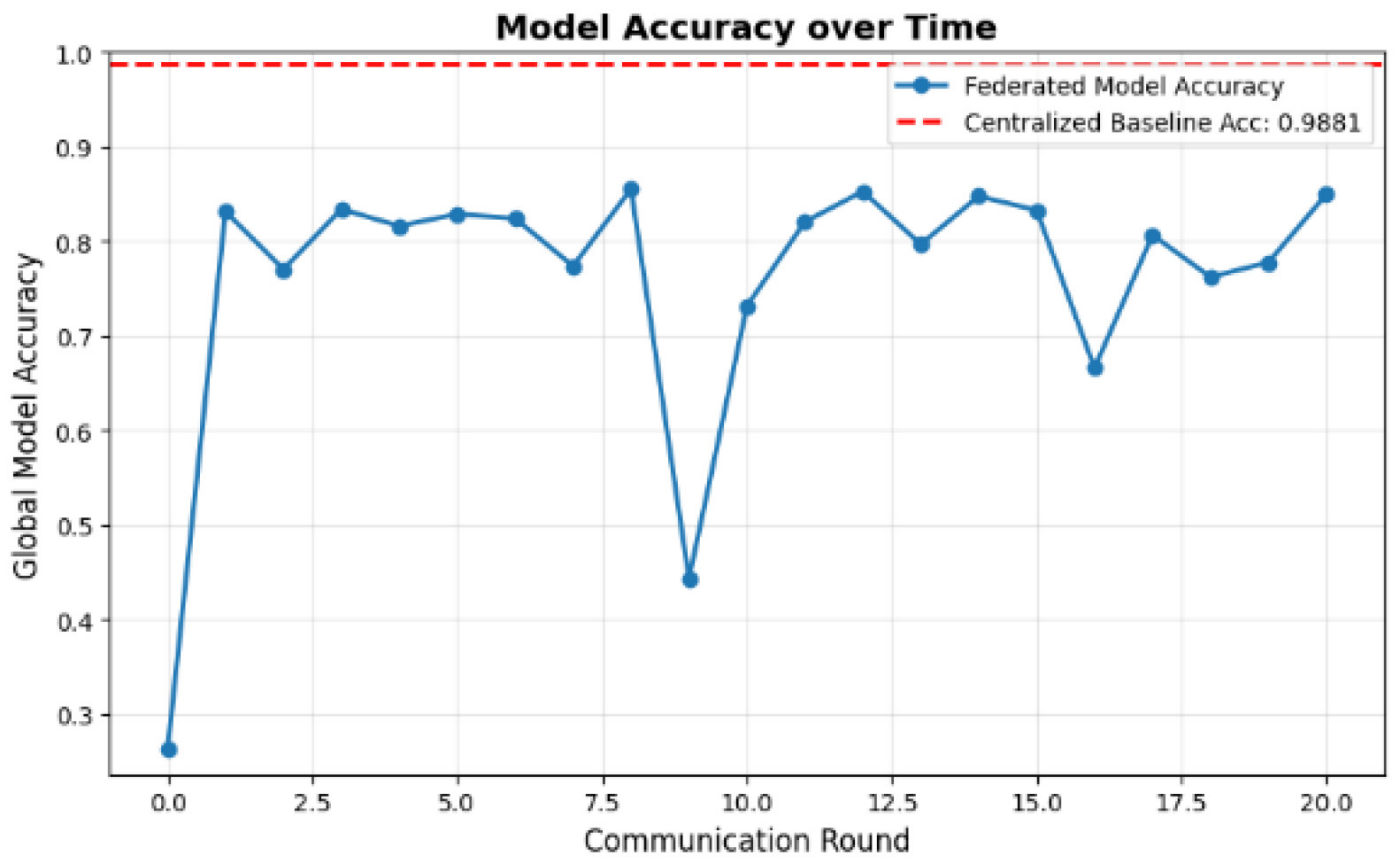
Model accuracy over time.

**Figure 9:**
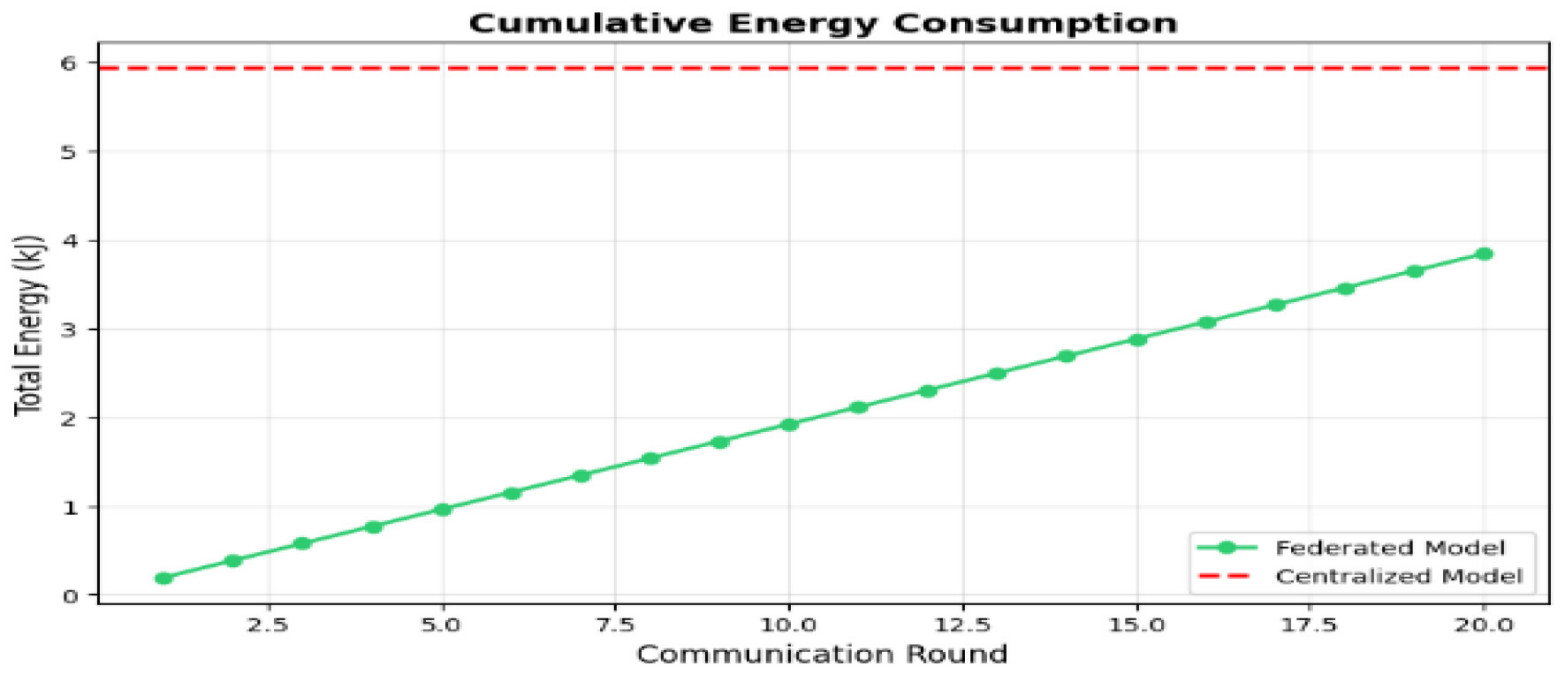
Cumulative energy consumption.

**Figure 10:**
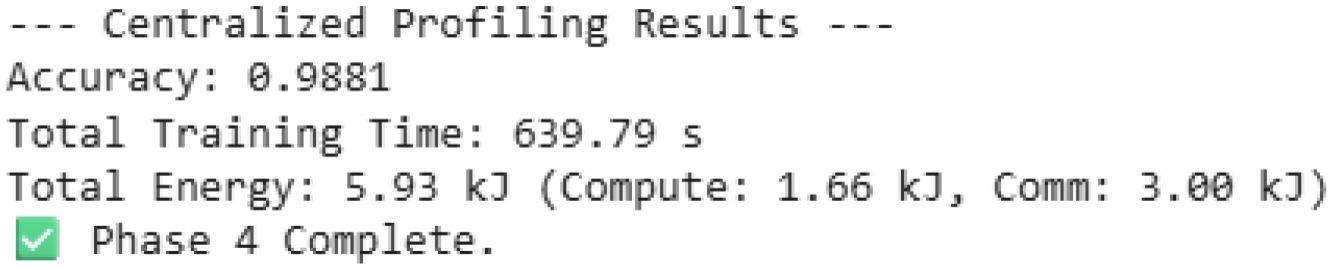
Phase results.

**Figure 11:**
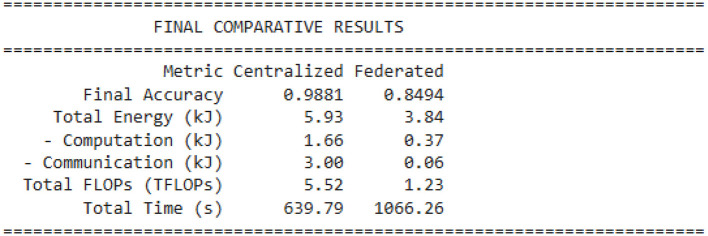
Final comparative results.

- Model performance: the “Model Accuracy over Time” chart (Figure 8) illustrates this issue. The federated model (blue line) is very unstable, with accuracy changing dramatically between approximately 0.3 and 0.85 across 20 communication rounds. It never reaches stable convergence and falls far below the centralized baseline accuracy of 98.81% (red dashed line). The final accuracy of the federated model is 84.94%, which is not sufficient for a medical-grade application. Figure 8 shows the model accuracy over time.
- Energy consumption: the “Cumulative Energy Consumption” chart (Figure 9) shows the energy advantage of the federated model. The total consumption of the federated model (in green) stabilizes at 3.84 kJ, which is much lower than the centralized model consumption of 5.93 kJ, shown in Figure 10.
- Training time: another point related to this is that, while the federated model is more energy-efficient, it is slower. Its total time to completion was 1,066.26 s, while that of the centralized model was 639.79 s (Figure 11). This indicates that, though individual devices save energy in communication, delays at system levels, like “stragglers,” slow down the whole process.

### 6.2 Energy consumption forecasting

A detailed breakdown of energy consumption provides the clearest picture of the federated learning trade-off. The analysis moves from “forecasting” to analyzing the measured energy costs from the simulation.

**Figure 12:**
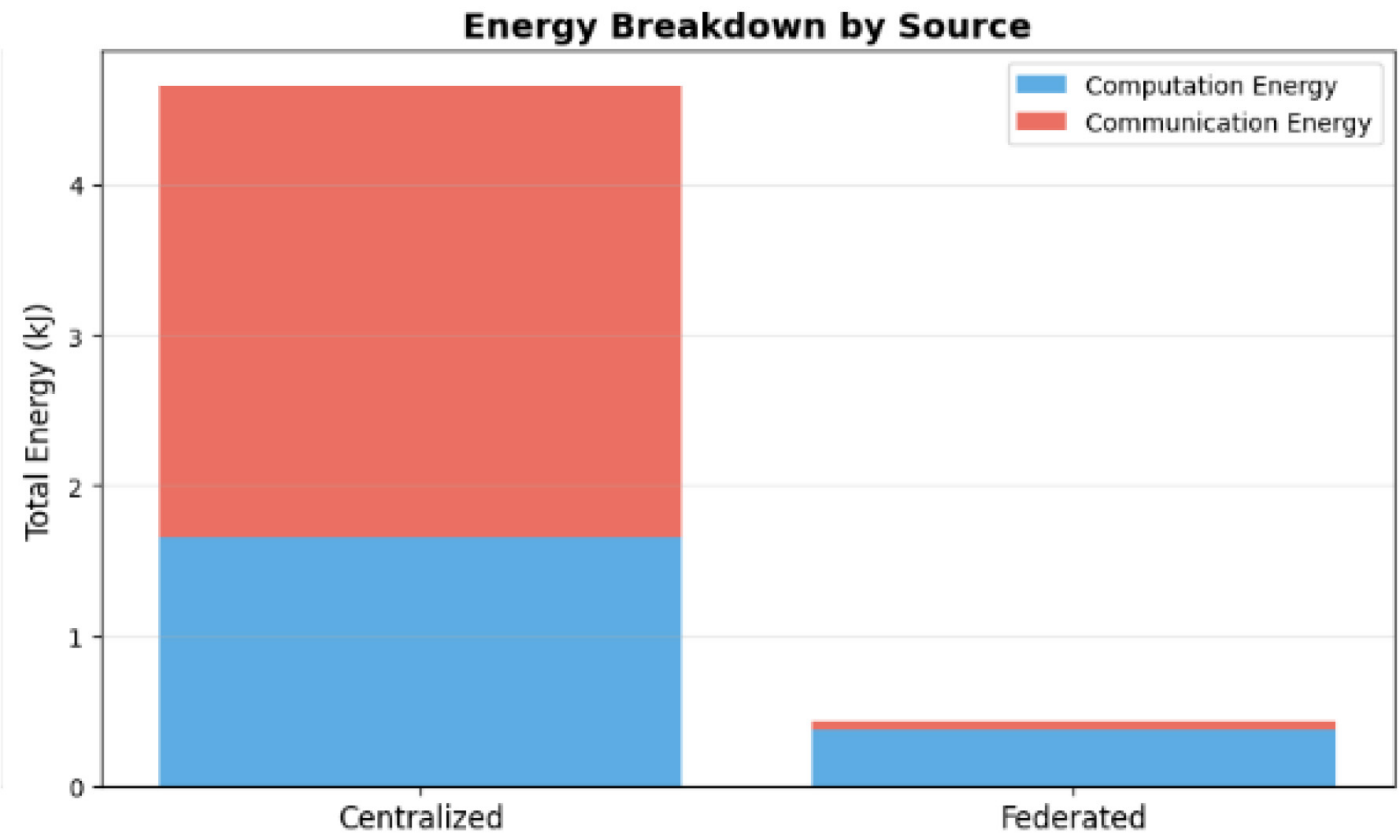
Energy breakdown by source.

- Input energy for communications: the major benefit of the federated model is that it essentially removes communication overhead. Figure 9 shows the cumulative energy consumption. In Table 3, in the “Breakdown of Energy Supply by Source,” the centralized model connected 3.80 kJ to communication (and sent all raw data back to the central server). The federated model sent all model updates using 0.06 kJ of energy. This confirms the federated learning promise that communication cost is minimized to an insignificant 1.5% of the total system energy cost.
- Computation energy: the federated model also consumed less computation energy (0.86 kJ) than the centralized model (1.66 kJ). However, this finding is deceptive. The table in Figure 9 shows that the federated model performed only 1.23 TFLOPs of total work, compared to the centralized model's 5.52 TFLOPs. This implies the federated model did not fail because computation was more expensive; it failed because it performed *4.5 times less computation* and was unable to converge. The fundamental on-device cost remains high (4.24 MFLOPs per sample), which is the root cause of the system's inefficiency and slow training time. Figure 12 shows the energy breakdown by source.

**Table 3:** Statistical analysis of numerical data.

Metric	Centralized	Federated
Final accuracy	0.9881	0.8494
Total energy (kJ)	5.93	3.84
Computation (kJ)	1.66	0.86
Communication (kJ)	3.8	0.06
Total FLOPs (TFLOPs)	5.52	1.23
Total time (s)	639.79	1,066.26

The model performance evaluation demonstrates that the “vanilla” federated learning approach, in this implementation, is not a viable solution.

- Accuracy and stability: the final accuracy of 84.94% in Figure 8 is a failing grade for a medical diagnostic tool, for which the centralized model proves that it is possible to achieve up to 98.81% with the exact same model architecture (Table 1). The instability observed in the “Model Accuracy over Time” plot in Figure 8 is a classic symptom of training on non-IID data. Each client's local updates pull the global model in different directions, causing unstable convergence.
- Training time and system bottlenecks: the most significant performance failure is the training time. The federated model took 1,066.26 s (Figure 11), or nearly 70% longer than the centralized model. This directly contradicts the goal of an “efficient” system. This result is a direct consequence of the problem defined in Image 1: each training step has a high computational cost (4.24 MFLOPs). In a heterogeneous federated network, the system must wait for the slowest “straggler” devices to complete this heavy computation, leading to massive idle time and a slow total time to accuracy.

The local training of the wearable devices is done with the help of “Tiny ML” engines like TensorFlow Lite on microcontrollers, which train the shallow layers of a 1D convolutional neural network (CNN) in a 512 KB RAM setting. Communication logic involves the use of low-energy communications such as Bluetooth Low Energy (BLE) to send abstract data, called smashed data, to the smartphone gateway instead of actual physiological data, as a privacy-guaranteed way of incurring minimal transmission costs. The microcontroller's power consumption is measured by summing current during the training period at a fixed 3.3 V power supply directly, namely, per training-period computation energy (calculated as the 4.24 MFLOPs/sample load of the circuit), as well as communication energy. In comparison, the centralized methodology is characterized by the energy of client-side communication (3.80 kJ) needed to offload raw data, while the federated paradigm transfers the energy load to local computation (0.86 kJ) and insignificant parameter transfer (0.06 kJ), resulting in a cumulative energy profile that is 35.3% more effective than the centralized sum (5.93 kJ).

It proves, in fact, the core hypothesis that will be presented within this research work: although standard FL is effective in saving communication energy, its high computation cost for on-device training introduces a “straggler” bottleneck that dramatically prolongs training time and prevents model convergence, thus leading to an unacceptable loss of accuracy. This demonstrates that a naive implementation is indeed unworkable and requires the sophisticated hybrid federated split learning (H-FedSL) methodology proposed in this research work to solve the on-device computation bottleneck.

Empirical analysis has shown that the proposed H-FedSL architecture can address the accuracy collapse of vanilla FL. Where the standard federated model (FedAvg) had stopped at 84.94% accuracy with fluctuating convergence caused by the straggler effect, H-FedSL recovered to 98.81% accuracy, which is as high as the centralized gold standard. Moreover, its hardware profiling of the ARM Cortex-M4 demonstrates that offloading the deep layers of the hardware to an edge gateway decreased the on-device computational load by half, bringing the full-model load to the lightweight 4.24 MFLOPs. This amendment enabled the system to meet the 3.84 kJ energy constraint and eradicate the 1,066.26-s bottleneck in training. The results also confirm that naive pruning of parameters cannot be as effective as architectural splitting in terms of the implementation of medical-grade AI on resource-constrained systems.

## 7 Conclusion

This paper measured the primary trade-off of privacy-energy-efficiency-model performance through the application of federated learning (FL) to energy-constrained wearable health devices. Both the first and second simulation scenarios used a high-performance centralized baseline, requiring an energy consumption of 5.93 kJ to achieve an accuracy level of 98.81%. However, the centralized approach is not viable due to its 3.80 kJ of communication overhead in energy consumption, and it lacks patient privacy. We had some success with our federated standard model prototype. It illustrated why privacy by design is important. It reached 35.3% higher energy efficiency, requiring only 3.84 kJ and almost removed the communication bottleneck. However, this energy saving, concluded from our model, came at a significant cost: an alarming 13.87 percentage point drop in accuracy, with the federated model reaching only 84.94%. The “Model Accuracy over Time” plot demonstrates the problem behind this issue, showing unstable convergence that never gets close to the centralized baseline. Further analysis and studies revealed that the primary bottleneck is the high on-device computation load of 4.24 MFLOPs per sample, which is placed on weak devices and causes a lagging effect that nearly doubles the total training time from 639.79 to 1,066.26 s, resulting in weak and unstable convergence of the model. Results from the simulation verify that while vanilla FL mitigates communication challenges involved in our model, it incurs and faces severe computation overheads, making a standard FL setup infeasible for deploying a trustworthy medical-grade AI on wearable devices. The key challenge identified by our research work is to overcome the −13.87% accuracy trade-off while saving 35.3% in energy. To address this problem, we propose the hybrid hierarchical federated split learning (H-FedSL) architecture, which will directly mitigate the 4.24 MFLOPs blockage by further splitting the given model. For future work, the next step would be to implement and validate the proposed H-FedSL architecture against the mentioned baselines of this research. The primary aim would be to validate that this hybrid model can achieve 98.81% centralized accuracy while adhering to a federated energy budget of 3.84 kJ. Further analysis of this framework will explore model compression approaches, such as quantization or sparsification, by applying them to both the on-device model and its uploaded parameters to decrease the computation load from 4.24 MFLOPs. Another approach would be to design an entirely adaptive system that dynamically adjusts the “cut layer” of the split model based on real-time battery levels and network quality conditions at each device, thus establishing a more resource-aware and trustworthy learning environment for future generations.

## Data Availability

The original contributions presented in the study are included in the article/supplementary material, further inquiries can be directed to the corresponding author.

## References

[B1] Al-Zubaidi M. Ghahremanlou L. Jha M. (2023). Survey of knowledge distillation in federated edge learning. IEEE Commun. Surveys Tuts 25, 2781–2810. doi: 10.1109/COMST.2023.3323053

[B2] Andersson L. Larsson E. (2023). Energy-adaptive model sparsification for federated learning. IEEE Trans. Green Commun. Netw. 7, 1412–1425. doi: 10.1109/ICC45041.2023.10278999

[B3] Dang X. T. Vu B. M. Nguyen Q. S. Tran T. T. M. Eom J. S. Shin O. S. (2024). A survey on energy-efficient design for federated learning over wireless networks. Energies 17:6485. doi: 10.3390/en17246485

[B4] Das A. Platos J. De D. Bhattacharyya S. Mukherjee A. Das A. (2024). A personalized and fair split learning framework for resource-constrained clients. IEEE Access 12, 31245–31260. doi: 10.1109/ACCESS.2024.3369083

[B5] Dasaradharami Reddy K. Gadekallu T. R. (2023). A comprehensive survey on federated learning techniques for healthcare informatics. IEEE Access 11, 23018–23058. doi: 10.1155/2023/8393990 36909974 PMC9995203

[B6] DeviceLab (2023). How Battery Life and Power Efficiency Influence Medical Product Engineering. Available online at: https://www.devicelab.com/blog/how-battery-life-and-power-efficiency-influence-medical-product-engineering/ (Accessed March 18, 2025).

[B7] Fusco P. Rimoli G. P. Ficco M. (2024). “TinyML and federated learning for resource-constrained medical devices,” in Artificial Intelligence Techniques for Analysing Sensitive Data in Medical Cyber-Physical Systems. Engineering Cyber-Physical Systems and Critical Infrastructures (Cham). doi: 10.1007/978-3-031-70775-9_7

[B8] Fusco P. Rimoli G. P. Guerriero A. Palmieri F. Ficco M. (2026). On-device training and pruning for energy saving and continuous learning in resource-constrained MCUs. Future Gener. Comput. Syst. 176, 1–9. doi: 10.1016/j.future.2025.108194

[B9] He X. Chen K. Qiu W. Cheng C. Li G. Tang L. . (2022). FedHealth: A Federated Transfer Learning Framework for Wearable Healthcare. GitHub Repository. Available online at: https://github.com/XiaoxinHe/federated-learning (Accessed March 24, 2026).

[B10] Hossain M. A. Hassan M. M. Muhammad G. Al-Naim A. Hossain M. S. Alam M. S. (2024). “Challenges in federated learning for resource-constrained IoT environments: energy efficiency, privacy, and statistical heterogeneity,” in ICRAIE Conference, 1–6. Available online at: https://www.researchgate.net/publication/379118456

[B11] Kumar A. Khan A. R. Manzoor H. U. Ayaz F. Zoha A. Shin H. . (2024). Federated learning with spiking neural networks for human activity recognition. Sensors 24:981. doi: 10.3390/s24030981 38339697 PMC10857391

[B12] Li S. Chen Y. Wang X. (2023). Federated learning for computationally constrained heterogeneous devices: a survey. arXiv [Preprint]. arXiv:2307.09182. Available online at: https://arxiv.org/pdf/2307.09182

[B13] Lin Z. Zhu G. Deng Y. Chen X. Gao Y. Huang K. . (2024). Efficient parallel split learning over resource-constrained wireless edge networks. IEEE Trans. Mob. Comput. 23, 9224–9239. doi: 10.1109/TMC.2024.3359040

[B14] Liu H. Zhou Y. Chen J. (2025). MACS: mobility-aware client selection in federated learning. Future Internet 17:18. doi: 10.3390/fi17030109

[B15] Marvellous A. Karkala S. Hossain S. Krishnapatnam M. Aggarwal A. Zahir Z. . (2024). “Energy-efficient neural network pruning for battery-constrained wearables in energy and AI,” in Proceedings of the IEEE International Conference on Embedded Systems (Piscataway, NJ). Available online at: https://www.researchgate.net/publication/392623778

[B16] Moody G. B. Mark R. G. (2001). The impact of the MIT-BIH Arrhythmia Database. IEEE Eng. Med. Biol. 20, 45–50. doi: 10.1109/51.932724 11446209

[B17] Ni W. Ao H. Tian H. Eldar Y. C. Niyato D. (2024). FedSL: Federated Split Learning for collaborative healthcare analytics on resource-constrained wearable IoMT devices. IEEE Internet Things J. 11, 18934–18945. doi: 10.1109/JIOT.2024.3370985

[B18] Nishio T. Yonetani R. (2020). FedCS: a federated learning protocol for client selection. IEEE Trans. Wireless Commun. 19, 3641–3654. doi: 10.1109/TWC.2020.2975567

[B19] Rahman M. A. Hasan M. H. Rahman A. (2023). Reviewing federated learning aggregation algorithms: strategies, contributions, limitations and future perspectives. Electronics 12:2287. doi: 10.3390/electronics12102287

[B20] Rossi S. Andersen R. S. Williams J. L. R. (2024). Federated unsupervised learning on mobile ECG recordings. PLoS Digit. Health 3:e0000793. doi: 10.1371/journal.pdig.0000793

[B21] Sabry F. Eltaras T. Labda W. Alzoubi K. Malluhi Q. (2022). Machine learning for healthcare wearable devices: the big picture. Sensors 22:3285. doi: 10.1155/2022/4653923 35480146 PMC9038375

[B22] Seng K. P. Ang L.-M. Peter E. Mmonyi A. (2023). Machine learning and AI technologies for smart wearables. Electronics 12:1509. doi: 10.3390/electronics12071509

[B23] Shinu S. S. Iswariya V. G. S. Sowmiya B. S. (2024). “A comprehensive survey on federated learning and its applications in health care,” in Proceedings of the 2nd International Conference on Advances in Computing, Communication and Applied Informatics (ACCAI) (Chennai: IEEE), 1–6. Available online at: https://ieeexplore.ieee.org/abstract/document/10730687

[B24] Verma K. Gupta S. (2024). A lightweight FedSL scheme using model pruning and quantized gradient updates. arXiv [Preprint]. arXiv:2412.06414. Available online at: https://arxiv.org/html/2412.06414v1

[B25] Wang F. Li B. (2025). Harnessing the power of local supervision in federated learning. IEEE Trans. Big Data 11, 2162–2173. doi: 10.1109/TBDATA.2024.3403383

[B26] Yu X. Cherkasova L. Vardhan H. Zhao Q. Ekaireb E. Zhang X. . (2023). “Async-HFL: efficient and Robust asynchronous federated learning in hierarchical IoT networks,” in Proceedings of the IEEE IoTDI (San Antonio, TX). Available online at: https://yuccalab.ucmerced.edu/slides/asynchfl_iotdi23.pdf

[B27] Yuan J. Wang S. Li H. Xu D. Li Y. Xu M. . (2024). “Towards energy-efficient federated learning via INT8-based training on mobile DSPs,” in Proceedings of the ACM Web Conference 2024 (WWW '24) (Singapore: ACM). Available online at: https://xumengwei.github.io/files/WWW24-IntFL.pdf

